# The possibility of endoscopic treatment of cN0 submucosal esophageal cancer: results from a surgical cohort

**DOI:** 10.1007/s00464-020-07420-y

**Published:** 2020-02-18

**Authors:** Bo Ye, Xiaobin Zhang, Yuchen Su, Shuguang Hao, Haohua Teng, Xufeng Guo, Yu Yang, Yifeng Sun, Teng Mao, Zhigang Li

**Affiliations:** 1 Department of Thoracic Surgery, Shanghai Chest Hospital, Shanghai Jiao Tong University, Huaihaixi Road 241, Shanghai, 200030 People’s Republic of China; 2 Department of Thoracic Surgery, Xinxiang Central Hospital, Xinxiang, 453000 Henan Province People’s Republic of China; 3 Department of Pathology, Shanghai Chest Hospital, Shanghai JiaoTong University, Huaihaixi Road 241, Shanghai, 200030 People’s Republic of China

**Keywords:** Esophageal, Squamous cell carcinoma, Endoscopic submucosal resection, Clinical N0 pathologic T1b

## Abstract

**Background:**

We analyzed the pathological characteristics and recurrence pattern of cN0 submucosal esophageal cancer after esophagectomy and conducted risk stratification to determine the feasibility of performing endoscopic resection for cN0pT1b esophageal squamous cell malignancies.

**Methods:**

We retrospectively enrolled 167 patients who underwent right-sided transthoracic esophagectomy and extended thoracic/abdominal two-field lymphadenectomy. Patients with pathologically confirmed lymph node metastasis or tumor recurrence constituted the high-risk group for endoscopic submucosal resection, and the remainder were defined as low risk. Factors affecting lymphatic metastasis and long-term recurrence were identified by univariate and multivariate analyses.

**Results:**

Postoperative pathology showed that five patients (5/167; 3%) had lymph node metastases. Follow-up ranged from 12–60 months, with a median of 29 months. A total of 17 patients (10.2%) had recurrences during follow-up, including three patients with pathologic nodal metastasis (pN +) found at surgery. Invasion depth, differentiation, and tumor size differed significantly in high-risk patients. Overall 3-year survival rates were 94.2% (low-risk) and 40.9% (high-risk) (*p* < 0.01). Twenty-one patients with sm1 cancer, high tumor differentiation, and tumor length < 2 cm had no lymph node metastasis or lymphovascular invasion, and none of these patients experienced recurrence.

**Conclusions:**

Endoscopic submucosal resection alone may be feasible for patients with small (≤ 2 cm) clinically N0 submucosal esophageal squamous cell carcinoma with low invasion depth (sm1) and higher differentiation, but prospective studies are required for confirmation. Other patients require surgical resection with extended two-field thoracic/abdominal lymphadenectomy.

Superficial esophageal cancer is defined as esophageal carcinoma with tumor invasion limited to the submucosa. Because of the wide use of endoscopic screening techniques, approximately 36% of patients with esophageal squamous cell carcinoma (SCC) are diagnosed with superficial lesions in Japan [1]. Improving overall treatment efficacy has become a key in enhancing overall outcomes in patients with esophageal SCC. Because the rates of lymph node metastasis with intramucosal (T1a) esophageal carcinomas are extremely low, endoscopic submucosal resection alone has become the standard treatment [1–3]. In contrast, T1b esophageal carcinomas with submucosal invasion have a much higher lymph node metastasis rate of approximately 12–54% [4–7]. Therefore, the standard treatment for submucosal lesions consists of radical resection of the esophageal cancer plus lymph node dissection.

Surgical treatment of esophageal cancer is traumatic for patients and associated with high postoperative complication rates [8]. Endoscopic submucosal resection is an alternative treatment for T1b patients unwilling to undergo radical resection [3]. A recent study by the Japan Clinical Oncology Group (JCOG0508 study) [9] of patients with T1b (sm1–2) N0M0 thoracic esophageal SCC confirmed the efficacy of endoscopic resection combined with selective chemoradiotherapy, with results comparable to those following surgery [9, 10]. However, patient selection in the JCOG0508 study was based on a main tumor depth of invasion of csm1–2 by endoscopic ultrasonography or pathological diagnosis from endoscopically resected specimens, which do not accurately reflect the depth of invasion, especially the possibility of sm3 involvement. Additionally, the sample size was not large (*n* = 86; pT1b). Larger numbers of surgical cases are needed to fully explain which patients are suitable for non-surgical treatment.

This retrospective study analyzed data for patients with pathologically confirmed T1b esophageal SCC with clinical N0 submucosal lesions treated with esophagectomy in our center over a 3-year period. We compared the different clinical features of high-risk and low-risk patients, where high-risk patients were those with surgical lymph node metastasis or later recurrence, and the remainder were defined as low-risk patients. The latter are considered possible endoscopic treatment candidates who can avoid esophagectomy and extensive lymph node dissection.

## Materials and methods

We retrospectively analyzed data from 2023 consecutive patients with primary esophageal SCC who were treated at Shanghai Chest Hospital from January 2014 to December 2017. Of these, 229 (11.32%) patients had pathologically confirmed esophageal SCC with submucosal invasion (pT1b).

**Fig. 1:**
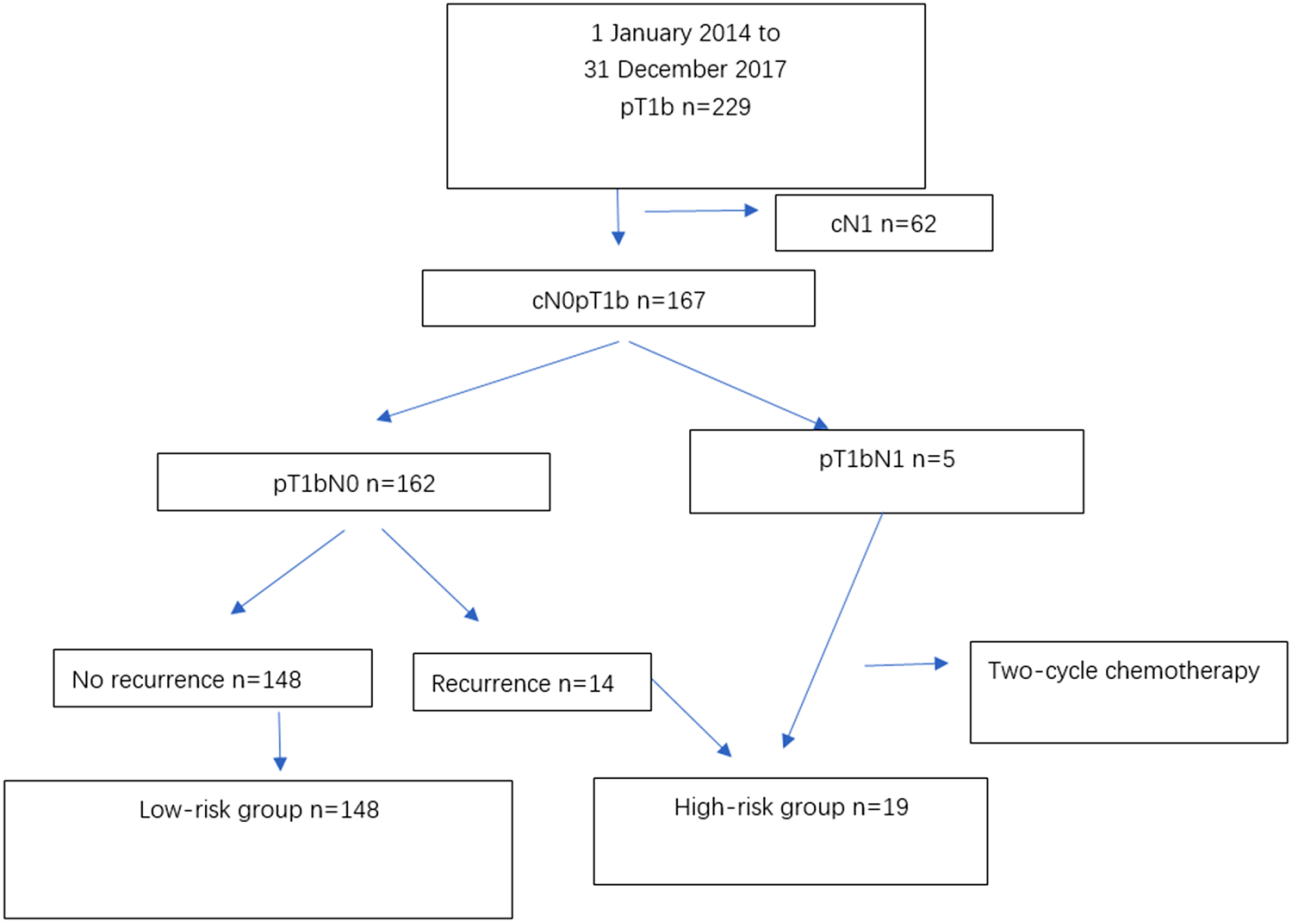
Flow chart showing the patient selection process for the study

After excluding 62 patients preoperatively diagnosed with lymph node metastasis (cN1), we analyzed data for 167 patients diagnosed with cN0pT1b esophageal SCC (Fig. 1). All 167 patients underwent R0 resection. Our focus in this study was patients with stage cN0pT1b esophageal SCC, therefore, we excluded data for stage cN0cT1b patients.

The study protocol was approved by the Shanghai Chest Hospital ethics committee, which waived the requirement for informed consent because of the retrospective design.

All patients underwent thoracic and abdominal lymph node dissection. We retrospectively reviewed and recorded patients’ general information, surgical procedures, postoperative hospitalization details, follow-up duration, and recurrence and survival rates after discharge from the hospital.

Preoperative assessment included contrast-enhanced thoracoabdominal computed tomography (CT), cervical ultrasound, and upper gastrointestinal endoscopy. All patients underwent endoscopic ultrasound, and only 53 patients underwent positive emission tomography.

### cN0 diagnostic criteria

Patients were diagnosed with cN0 disease if carotid/abdominal ultrasound, endoscopic ultrasound, contrast-enhanced thoracoabdominal CT, and other imaging modalities found no evidence of lymph nodes > 1 cm diameter. Other diagnostic criteria for cN0 disease included an absence of central liquefaction/necrosis, peripheral enhancement, or disappearance of the fatty gap near the lymph nodes. No patients underwent endoscopic ultrasound-guided biopsy to confirm cN0 status.

### Surgical treatment

Decisions on appropriate therapy for superficial esophageal SCC were discussed by a multidisciplinary tumor board. No patients received preoperative induction therapy, and all patients underwent thoracic and abdominal lymph node dissection, including dissection of the lymph nodes near bilateral recurrent laryngeal nerves.

### Postoperative pathological diagnosis

Pathological sections for all clinically diagnosed T1b esophageal SCC samples were retrospectively analyzed by two senior pathologists. T1b (submucosal invasion) was defined as invasion into the submucosal layer but not reaching the muscularis propria. The specific pathological characteristics that we analyzed were tumor length, location, differentiation, and depth of invasion. Depth of invasion was categorized as: sm1, invasion of the upper third of the submucosal layer or ≤ 200 μm from the mucosal muscularis in the specimens resected by endoscopic resection; sm2, invasion reaching the middle third of the submucosal layer; or sm3, invasion reaching the lower third of the submucosal layer. Other parameters were lymphovascular invasion, perineural invasion, total number of lymph nodes dissected, and number and location of positive lymph nodes.

We recorded the concordance rate between the two senior pathologists, and differences in opinion regarding the pathological diagnosis for any sample were resolved by discussion among all of the department's pathologists.

### Follow-up

Patients were followed at the out-patient clinic or by telephone 1, 3, 6 months, and then every 6 months, postoperatively. Esophagogastroduodenoscopy, and CT of the thorax, abdomen, and neck were routinely performed during each follow-up examination, and we used the Clavien–Dindo system to evaluate complications [11].

### Statistical analysis

All statistical analyses were performed using SPSS software (IBM SPSS Statistics, V21, macOS X; IBM Corp., Armonk, NY). Categorical data were analyzed with the Chi-square test. For continuous data, we used the independent *t* test or Mann–Whitney U test to analyze age, depth of invasion, and tumor length. We also performed multivariate logistic regression analysis using a backward stepwise procedure and adding all of the significant risk factors from the univariate analysis to identify the significant predictors for the high-risk group. Survival was defined as the time from the first postoperative day to the last day of follow-up or death. We used Kaplan–Meier survival curves to calculate recurrence-free and overall survival for all patients, and *p* < 0.05 was considered statistically significant.

## Results

We analyzed data for 167 patients, namely, 131 men (81.4%) and 36 women (21.6%) with a mean (± standard deviation) age of 62.9 ± 8.8 years. Of these patients, 22 (13.2%) had lesions in the upper third, 111 (66.5%) in the middle third, and 34 (20.4%) in the lower third of the esophagus. Preoperative T stages included T1a in 30 patients (18%), T1b in 106 (63.5%), T2 in 31 (18.5%), and T3 in no patients; all patients were preoperative N stage 0 (N0).

No patients received preoperative induction therapy. All patients underwent esophageal SCC resection via a right transthoracic approach, with 151 (90.4%) undergoing McKeown (three-incision) esophagectomy and 16 (9.58%) undergoing Ivor–Lewis operations; 136 patients (81.4%) underwent minimally invasive video-assisted thoracoscopy, and 31 (18.6%) underwent open surgery.

Complications of any type occurred in 41.3% (69/167) of the patients. The percentage of patients experiencing Clavien–Dindo complications ≥ grade 3 was 12% (20/167). The main complications were anastomotic leak (confirmed radiographically) in 28 patients (16.7%), respiratory insufficiency in 10 (6%) (defined as patients requiring reintubation or a non-invasive ventilator support), and recurrent laryngeal nerve paralysis (confirmed using laryngoscopy 1-week postoperatively) in 19 (11.4%) patients (Table 1).

**Table 1:** Patients' demographic and clinical characteristics (*n* = 167)

Variable	*n*(%)
Age (years) ± standard deviation	62.91 ± 8.80
Sex	
Male	131(78.44)
Female	36(21.56)
Tumor location in the esophagus	
Lower third	34(20.36)
Middle third	111(66.47)
Upper third	22(13.17)
Tumor length (mm)	20.36 ± 10.18
Surgical approach	
McKeown	151(90.42)
Ivor–Lewis	16(9.58)
Minimally invasive esophagectomy	136(81.44)
Open	31(18.56)
Complications	
Anastomotic leak	28(16.77)
Respiratory failure	10(5.99)
Recurrent laryngeal nerve paralysis	19(11.38)
In-hospital Stay (days) (mean, range)	18.50(7–190)
Death within 90 days postoperatively	0(0)

*n* number

The average hospital stay was 18.5 days (range, 7–190 days). No patients died in-hospital or within 90 days postoperatively (Table 1), and 5 patients with lymph node metastasis received two cycles of adjuvant chemotherapy.

Postoperative pathological examination showed submucosal invasion (T1b) in all patients. The numbers of patients with sm1, sm2, and sm3 invasion were 96/167 (57.5%), 48/167 (28.7%), and 23/167 (13.8%). The median number of dissected lymph nodes was 23 (range, 10–44). Postoperative pathology showed that five patients (5/167, 3%) had lymph node metastases, which were located at the thoracic paratracheal (*n* = 3, 60%), paracardiac (*n* = 1, 20%), and subcarinal (*n* = 1, 20%) sites. Of the patients with sm1, sm2, and sm3 invasion, 0, 2/48 (4.2%), and 3/23 (13.04%), respectively, had lymph node metastases (*p* < 0.05, for all comparisons). Lymphovascular invasion was detected in 10 patients, but none had postoperative lymph node metastasis or recurrence.

The 19 patients (19/167, 11.4%) with pathologically confirmed lymph node metastasis or tumor recurrence were classified as the high-risk group, namely, 5 patients with positive lymph node metastasis and 14 patients with recurrent disease, with the remaining 148 patients (148/167, 88.6%) classified as the low-risk group. During follow-up, 17 patients (17/167, 10.2%) developed recurrence at a median of 683.5 days, namely, 14/162 (8.6%) in the pN0 group and 3/5 (60%) in the pN+ group. Of the 14 patients with recurrent tumors in the pN0 group, 12 were classified as sm2 or sm3, 13 had moderate/low differentiation, and 13 had a diameter ≥ 2 cm. Of the 17 patients with tumor recurrence (14 in the pN0 group and 3 in the pN+ group), 9 (52.9%) had recurrent tumors in the neck/superior mediastinum, 5 (29.4%) in the carina/left and/or right bronchus, 3 (17.6%) in the inferior mediastinum/abdomen, and 1 (5.8%) in the pleural cavity (pleural effusion).

Univariate analysis showed that degree of differentiation, invasion depth, and tumor length differed significantly in the two groups (*p* < 0.05 for all comparisons) (Table 2). Multivariate analysis showed that degree of differentiation, invasion depth, and tumor length were independently associated with the likelihood of being in the high-risk group (Table 3).

**Table 2:** Univariate analyses of demographic and clinical characteristics of low- and high-risk patients with clinical N0 pathological T1b esophageal squamous cell carcinoma

Variable	Low-risk group (*n* = 148)	High-risk group (*n* = 19)	*p* value^a^
Sex			0.067
Male	113(76.35)	18(94.74%	
Female	35(23.65)	1(5.26)	
Age	63.02 ± 7.3	62.05 ± 8.52	0.946
Surgical approach			
McKeown	135(91.22)	16(84.21)	0.329
Ivor–Lewis	13(8.78)	3(15.79)	
Minimally invasive esophagectomy	122(82.43)	14(73.68)	0.356
Open	26(17.57)	5(26.32)	
Number of LND	24.48 ± 7.90	23.11 ± 6.60	0.504^a^
Tumor location in the esophagus			0.904
Upper third	18(12.16)	3(15.79)	
Middle third	97(65.54)	12(63.16)	
Lower third	33(22.3)	4(21.05)	
Differentiation			< 0.001
Well	70(47.3)	2(10.53)	
Moderate	41(27.7)	5(26.32)	
Poor	37(25)	12(63.16)	
Depth of invasion			< 0.001
sm1	93(62.84)	3(15.79)	
sm2	43(29.05)	5(26.32)	
sm3	12(8.11)	11(57.89)	
LVI	10 (6.76)	0	0.243
Tumor length (mm)	17.18 ± 10.53	23.71 ± 6.85	< 0.001^a^
< 20 mm	81(54.73)	1(5.26)	
≥ 20 mm	67(45.27)	18(94.73)	

Values are presented as number, number(percent), or mean ± standard deviation

^a^Mann–Whitney U test; *LND* number of lymph nodes dissected, *LVI* lymphovascular invasion, *sm1* invasion of the upper third of the submucosal layer or reaching the submucosal layer > 200 µm from the muscularis, *sm2* invasion reaching the middle third of the submucosal layer, *sm3* invasion reaching the lower third of the submucosal layer

**Table 3:** Multivariate analysis of factors predictive of low risk (negative lymph node metastasis and recurrence-free survival) following endoscopic submucosal resection for esophageal squamous cell carcinoma

Variable	OR	95% CI	*p* value
Differentiation	0.138	0.085–0.524	< 0.001*
Depth of invasion (sm1)	0.246	0.115–0.634	< 0.001*
Tumor length (< 20 mm)	1.085	1.142–1.231	< 0.001*

*CI* confidence interval, *OR* odds ratio, *sm1* invasion of the upper third of the submucosal layer or reaching the submucosal layer > 200 µm from the muscularis

**Fig. 2:**
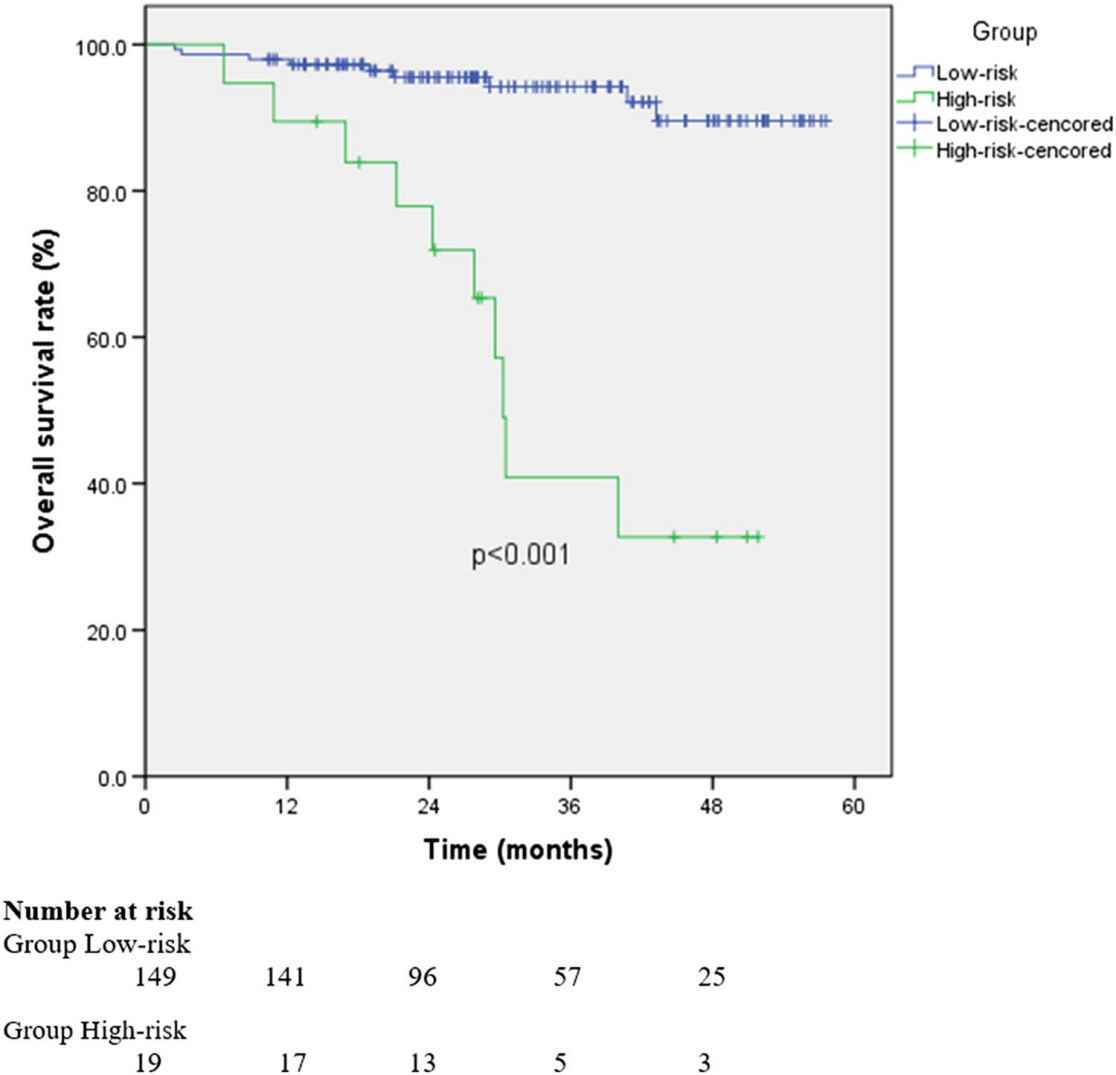
Overall survival in low- and high-risk patients with clinical N0 pathological T1b esophageal squamous cell carcinoma after endoscopic submucosal resection. *OS* overall survival

The median postoperative follow-up duration for all patients was 29 months. The median follow-up was 36.4 months (range, 13.5–51.8 months) in the high-risk group and 31.2 months (range, 10.4–57.6 months) in the low-risk group. Overall 3-year survival rates were 94.2% (low-risk group) and 40.9% (high-risk group) (hazard ratio: 9.418, 95% confidence interval: 3.819–23.222; *p* < 0.01) (Fig. 2).

Nineteen patients died after 90 days; 10 in the high-risk group and 9 in the low-risk group. In the high-risk group, 9 patients died of recurrence, and 1 died of abdominal infection. In the low-risk group, 1 patient died of suicide, 1 of heart disease, 3 of lung infection, 1 secondary to anastomosis stenosis and malnutrition, and 3 of unknown causes.

Regarding the diagnostic thresholds for endoscopic submucosal resection, a separate analysis showed that 21 patients with sm1 tumors, high tumor differentiation, and tumor length < 2 cm had no lymph node metastasis or lymphovascular invasion, and none experienced recurrence. Of the remaining 146 patients, 5 (5/146, 3.4%) experienced lymph node metastasis (*p* < 0.01), 17 (17/146, 11.6%) experienced recurrence (*p* < 0.01), and 10 (10/146, 6.8%) experienced lymphovascular invasion (Table 4). All of these 146 patients had at least one of the following findings: > sm1 tumors, low tumor differentiation, and tumor length > 2 cm.

**Table 4:** Differences between G_1_S_<20_ and G_2–3_S_>20_ patients

	Sm_1_ G_1_S_<20_ (*n* = 21)	Others (*n* = 146)
Lymph node metastases	0	5
LVI	0	10
Recurrence	0	17

*n* number, *G*_*1*_ high differentiation, *G*_*2–3*_*S*_>*20*_ low differentiation and tumor length > 20 mm, *LVI* lymphovascular invasion, *sm1* invasion of the upper third of the submucosal layer or reaching the submucosal layer > 200 µm from the muscularis, *S*_<*20*_ tumor length < 20 mm

The concordance rate between the two senior pathologists analyzing the tissue samples was 89.5% (205/229 histological diagnosis agreements).

## Discussion

The high incidence of complications after surgery for esophageal cancer remains a challenge. In most large-scale studies, the incidence of postoperative complications was approximately 40% [8]. Although advances in minimally invasive resection of esophageal SCC have improved patients’ postoperative quality of life and decreased the incidence of pulmonary complications, overall mortality rates have not changed dramatically [12, 13]. Radical treatment for esophageal SCC includes complete removal of the primary tumor and thorough lymph node dissection or inactivation. Endoscopic submucosal dissection can achieve R0 resection of some localized superficial lesions; however, because of the high rate of lymph node metastasis with submucosal esophageal carcinoma, no consensus has been reached regarding endoscopic submucosal resection of submucosal esophageal cancers [14]. Our retrospective analysis of patients with clinical N0 submucosal esophageal SCC identified a low-risk subgroup and provided diagnostic thresholds for endoscopic treatment in these select T1b patients.

The biological features of clinical N0 submucosal esophageal SCC are key to determining the feasibility of endoscopic treatment. However, less is known about the biological features of cN0pT1b esophageal SCC. Some studies evaluated small numbers of patients with esophageal adenocarcinoma [15, 16], while others included larger numbers and evaluated patients undergoing endoscopic submucosal resection for either T1a or T1b superficial esophageal adenocarcinoma [4, 17–20]. Two studies evaluating larger numbers of T1b patients did not evaluate cN0pT1b patients, specifically [5, 7]. More importantly, these studies did not include subgroup analyses. Because radical endoscopic treatment targets cN0 patients, oncological studies in patients with cN0 lesions can help determine the feasibility of endoscopic treatment.

Esophageal endoscopic ultrasonography (EUS) is generally considered the best tool for staging superficial esophageal lesions. In this study, all patients underwent EUS evaluation preoperatively; however, the diagnostic accuracy of T1b was only 63.5%, preoperatively. Diagnosing the N stage of superficial esophageal cancer appears to be more feasible. We found that the rate of lymph node metastasis in all T1b patients was as high as 29% (67/229) when cN+ was included. However, after preoperative CT and ultrasound evaluation, the postoperative pathological metastasis rate decreased to 3% (5/167) in the cN0 population. Thus, appropriate N staging can provide sufficiently accurate lymph node results and guide subsequent treatment. It is difficult to obtain a reference value for T staging. Endoscopic resection can be used both in the primary tumor diagnosis and in the treatment regime for superficial lesions.

To our knowledge, our study is the largest single-center study of patients with cN0pT1b esophageal SCC (*n* = 167). Because the overall lymph node metastasis rate in our patients (cN0pT1b) was 3% (5/167), R0 resection in patients with cN0pT1b lesions based only on clinical N0 findings is insufficient. Therefore, it is extremely important to identify additional patients at high risk of lymph node metastasis and recurrence. Our study indicated that the degree of tumor differentiation, invasion depth, and lesion size were risk factors for pathological lymph node metastasis and recurrence. Subgroup analysis showed that lymph node metastasis rates were 0, 1.4% (1/72), and 1.2% (1/82) in patients with sm1, highly differentiated, and smaller (< 2 cm) tumors, respectively; findings similar to those observed in patients with intramucosal (T1a) esophageal SCC [6]. Although lymph node involvement has been reported in up to 27% of patients with sm1 tumors, results may have been affected by the number of included patients [15]. Therefore, patients with sm1, highly differentiated, and small (< 2 cm) tumors are more likely to benefit from endoscopic R0 resection, alone. Our separate analysis showed that no patients with sm1, high tumor differentiation, and tumor length < 2 cm experienced lymph node metastasis, and no patients experienced recurrence, indicating these measures as diagnostic thresholds for endoscopic submucosal resection. Therefore, simple endoscopic submucosal resection may be sufficient for superficial lesions with sm1 invasion, high differentiation, and tumor length < 2 cm, which were the diagnostic thresholds for extended endoscopic treatment in these select T1b patients identified in our study. In contrast, surgical resection with extended thoracic and abdominal lymph node dissection remains the optimal treatment for high-risk patients with submucosal esophageal SCC.

Postoperative recurrences, particularly micrometastases, provide important clues for analyzing the scope of tumor invasion. Although our patients underwent radical thoracic and abdominal lymph node dissection, 8.4% (14/167) of patients experienced disease recurrence in the pN0 group. In this study, we classified patients with lymph node metastasis and/or distant recurrence as the high-risk group, and all others as the low-risk group. Univariate and multivariate analyses showed that degree of differentiation, invasion depth, and tumor length differed significantly between these two groups. Only 21/167 patients (12.6%) in our study met all three conditions for our diagnostic thresholds for endoscopic submucosal resection (i.e., sm1 invasion, high differentiation, and tumor length < 2 cm). These 21 patients experienced no lymph node metastasis or recurrence, therefore, endoscopic submucosal dissection with negative resection margins may be an appropriate treatment for inoperable low-risk patients meeting all three of the above conditions.

No consensus has yet been reached regarding treatment after endoscopic submucosal resection in patients with N0 high-risk submucosal esophageal SCC (> sm1, low degree of differentiation and tumor length > 2 cm). The adequacy of adjuvant chemoradiotherapy as a salvage treatment has not been well-documented [3, 9, 21]; however, our results suggest that salvage adjuvant chemoradiotherapy may be inadequate. First, most of the metastatic lymph nodes in our patients were distributed near the superior mediastinum or recurrent laryngeal nerve (3/5, 60%) and gastric cardia/left gastric artery (1/5, 20%). These findings were consistent with metastases of the submucosal esophageal SCC upward and downward following the long axis of the esophagus [22]. After endoscopic submucosal dissection, adjuvant radiotherapy alone in the area of the primary lesion cannot completely cover both areas, and extensive irradiation in multiple fields may increase toxicity. Furthermore, adjuvant chemotherapy has a limited role in controlling lymph node metastases in esophageal SCC. These findings suggest that surgical esophageal resection plus radical thoracic and abdominal lymph node dissection may be more effective in controlling these tumors. However, the results of the JCOG0508 study showed excellent long-term outcomes for T1b esophageal cancer treated by endoscopic resection followed by chemoradiotherapy. Therefore, endoscopic resection followed by chemoradiotherapy is now an important treatment strategy for T1b patients with clinical N0 esophageal SCC [10]. As a limitation, the JCOG0508 study involved only 86 (48.9%) pT1b patients and no sm3 patients. Our study involved 167 patients with clinical N0 pathologic T1b esophageal SCC confirmed surgically and 23 (23/167, 13.8%) sm3 patients. Our study is a good supplement to the JCOG0508 study, but studies involving more patients are needed to confirm our results. Additionally, prospective studies are needed to clarify which patients could benefit from endoscopic resection followed by esophagectomy.

Postoperative pathology showed that five patients had lymph node metastases: three in the left/right recurrent laryngeal nerve/superior mediastinum, one in the gastric cardia/peritoneum, and one in the carina/left or right bronchus. The three patients with recurrent laryngeal nerve/superior mediastinal metastasis constituted 60% of our patients with metastasis, which is a concern in the West, where dissecting these lymph nodes is rarely performed, even in patients with advanced disease. Rates of lymph node recurrence near the gastric cardia and around the celiac trunk were lower than in the superior mediastinum, showing that surgical treatment effectively controlled metastasis to these regions. Therefore, esophagectomy plus thorough thoracic and abdominal lymph node dissection, followed by small T-shaped field adjuvant radiation therapy in the neck and superior mediastinum, may be a more reasonable option for high-risk patients.

In this study, the overall incidence of postoperative complications was 41.3%, and involved anastomotic leak in 16.7% and respiratory insufficiency in 6% of the patients. No patients died within 90 days postoperatively, and the 3-year overall survival rate for the low- and high-risk groups, inclusive, was 86.9%. Therefore, surgery yielded good early outcomes and long-term survival indicating that, for patients with high-risk submucosal esophageal SCC, endoscopic treatment, even when followed by adjuvant chemoradiotherapy, was not as effective in controlling tumors as there was a surgical resection with extended thoracic and abdominal lymph node dissection, and postoperative cervical and upper mediastinal adjuvant radiotherapy. These survival results support using our diagnostic thresholds for extended endoscopic treatment in our defined low-risk T1b group.

This study has several limitations, namely, its single-center, retrospective design, and the lack of accurate clinical staging, which meant we were unable to compare outcomes of surgical vs non-surgical treatments in our patients with cN0pT1b esophageal SCC. Additionally, the number of sm1 patients was low. Another limitation is the cN0 classification without staging using positron emission tomography, therefore, some cN0 patients may have been understaged in our study. Additionally, no cN0 patients had lymph nodes biopsied by endoscopic ultrasonography/fine needle aspiration, which could also have affected accurate clinical lymph node staging.

In conclusion, careful clinical stratification of diagnosis and treatment is required in the treatment of patients with cN0 submucosal esophageal SCC. Simple endoscopic submucosal resection may be sufficient for superficial lesions with sm1 invasion, high differentiation, and tumor length < 2 cm, which were the diagnostic thresholds for extended endoscopic treatment in the select T1b patients identified in our study. More study is required, specifically randomized studies, to make any firm conclusions about endoscopic mucosal resection in this subset of patients. In contrast, surgical resection with extended thoracic and abdominal lymph node dissection remains the optimal treatment for high-risk patients with submucosal esophageal SCC. If lymph node metastasis is present, postoperative adjuvant treatment targeting the neck and superior mediastinum may prevent tumor recurrence.
